# Study on risk factor analysis and model prediction of hyperuricemia in different populations

**DOI:** 10.3389/fnut.2024.1417209

**Published:** 2024-10-14

**Authors:** Kaifei Hou, Zhongqi Shi, Xueli Ge, Xinyu Song, Congying Yu, Zhenguo Su, Shaoping Wang, Jiayu Zhang

**Affiliations:** ^1^Binzhou Medical University, Yantai, China; ^2^Laboratory Department, Yantai Affiliated Hospital of Binzhou Medical University, Yantai, China; ^3^The Second Hospital, Cheeloo College of Medicine, Shandong University, Jinan, China; ^4^School of Pharmacy, Binzhou Medical University, Yantai, China; ^5^School of Traditional Chinese Medicine, Binzhou Medical University, Yantai, China

**Keywords:** hyperuricemia, RF, SVM, risk factors, prediction model

## Abstract

**Objectives:**

The purpose of the present study was to explore the influencing factors of hyperuricemia (HUA) in different populations in Shandong Province based on clinical biochemical indicators. A prediction model for HUA was constructed to aid in the early prevention and screening of HUA.

**Methods:**

In total, 705 cases were collected from five hospitals, and the risk factors were analyzed by Pearson correlation analysis, binary logistic regression, and receiver operating characteristic (ROC) curve in the gender and age groups. All data were divided into a training set and test set (7:3). The training set included age, gender, total protein (TP), low-density lipoprotein cholesterol (LDL-C), and 15 other indicators. The random forest (RF) and support vector machine (SVM) methods were used to build the HUA model, and model performances were evaluated through 10-fold cross-validation to select the optimal method. Finally, features were extracted, and the ROC curve of the test set was generated.

**Results:**

TP, LDL-C, and glucose (GLU) were risk factors for HUA, and the area under the curve (AUC) value of the SVM validation set was 0.875.

**Conclusion:**

The SVM model based on clinical biochemical indicators has good predictive ability for HUA, thus providing a reference for the diagnosis of HUA and the development of an HUA prediction model.

## 1 Introduction

Hyperuricemia (HUA) is a metabolic disorder due to abnormal purine metabolism that results in elevated serum uric acid (SUA) levels (1). Approximately two-thirds of SUA in the human body is produced endogenously, while the remaining one-third is obtained from the diet (2). SUA levels are the result of the balance among uric acid (UA) biosynthesis in the liver, UA reabsorption in the proximal tubules of the kidney, and UA secretion from renal tubules and intestines. Approximately 70% of SUA in the human body is excreted through the kidneys, and the remaining 30% is excreted through the intestines and biliary tracts (3). As a public health problem, HUA is closely associated with the development of gout and is an independent risk factor for type 2 diabetes mellitus (T2DM), dyslipidemia, liver dysfunction, kidney disease, and metabolic syndrome, seriously jeopardizing human health (4).

SUA level is closely related to hyperglycemia and hyperlipidemia, and it can cause damage to liver and kidney function (5). The HUA observed in patients with kidney disease is thought to be caused by inadequate excretion of UA due to renal failure (6). A cross-sectional study of HUA in Chinese adults has indicated that central obesity, hyperlipidemia, hypertension, and low glomerular filtration rate are risk factors for HUA. In addition, Hou et al. (7) reported that triglycerides (TGs), total cholesterol (TC), and gender are closely associated with the occurrence of HUA. A previous study demonstrated that the risk of hypertriglyceridemia in HUA patients was approximately twice as high as in healthy individuals. Increased TGs led to increased production of free fatty acids and accelerated breakdown of ATP, resulting in increased UA (8).

As people’s living standards improve, the prevalence of HUA is rising rapidly in China. The lifestyles of Chinese individuals have dramatically changed, with increased intake of meat, dairy products, and other high-purine foods (9). In addition, a cross-sectional survey has reported that the overall prevalence of HUA in the Chinese adult population was 11.1% in 2015–16, with an alarming increase of 14.0% in 2018–19. Moreover, HUA tends to have a younger onset in both males and females (10). Furthermore, high SUA levels may result in significant financial losses to patients, including direct (e.g., medical consultation fees) and indirect (e.g., lost productivity) costs. The cost of screening gout patients increases with increasing SUA levels, and two-thirds of patients newly diagnosed with gout have not received uric acid-lowering therapy. Currently, musculoskeletal ultrasound (US) and dual-energy computed tomography (DECT) have been used as diagnostics for gout in a non-invasive manner. Compared with US, DECT has increased accuracy in the diagnosis of gouty arthritis (11, 12). However, we prefer to control the source of UA elevation, target HUA high-risk individuals in advance, and adjust their diets and lifestyles. Through scientific prevention and management, we may reduce the risk of HUA and gout and alleviate the pain caused by the disease.

Studies have showed that HUA was more prevalent in males than in females. In 2018–2019, the prevalence of HUA in China was 24.4% in males and 3.6% in females (10). A univariate analysis showed that the risk of HUA in males was 1.86 times higher than that of females (7). The lower prevalence of HUA in females may be caused by the ability of estrogen in females to regulate the level of the UA transporter in the kidneys through gene expression, thus reducing UA production and promoting its excretion. After menopause, estrogen levels in females decline so markedly that both genders tend to display no obvious differences (13, 14).

Machine learning has advantages of high accuracy and speed. It is of great significance to establish disease prediction models to assist in the diagnoses of diseases and reduce medical costs. In recent years, as the disease burden of HUA has increased, HUA prediction models have been gradually developed. Shi et al. (13) used the classification and regression tree (CART) algorithm to construct a model using four characteristics, namely, gender, age, body mass index (BMI), and hypertension; the model had a sensitivity of 0.87, specificity of 0.59, and an area under the curve (AUC) value of 0.80. Cao et al. (15) used Cox proportional hazards regression models incorporating age, body mass index (BMI), systolic blood pressure, serum UA and TGs for prediction. The *C* statistics was 0.782 for males and 0.783 for females. However, due to the small number of features included in the model, the fitting effect of the model was not perfect. Therefore, this study used biochemical indicators that was simple to obtain and covered a wide range to develop a more accurate and easily generalisable predictive model for HUA.

Clinically, HUA has an insidious onset, and most patients tend to be asymptomatic and have no clear biochemical markers, except for SUA. Because the factors involved in the pathogenesis of HUA are not well understood, the variables used in different studies have widely varied, resulting in inconsistent conclusions. Therefore, it is important to screen the risk factors affecting HUA and establish a prediction model for early identification, control, and intervention of HUA patients to reduce disease burden. The present study analyzed the correlation of HUA based on a cross-sectional study of data collected from five hospitals in Shandong Province to investigate the risk factors of HUA at different ages and genders to predict HUA through machine learning, providing an important reference for the development of regional health education and the formulation of accurate and scientific prevention and control strategies.

## 2 Materials and methods

### 2.1 Study participants

Data for this cross-sectional study were collected from five hospitals in four cities in Shandong Province, encompassing both inland and coastal cities. A total of 356 healthy volunteers (HC group) and 349 patients with HUA (HUA group) were included in the present study. According to the Practice Guidelines for the Diagnosis and Management of Hyperuricemia, the HUA group was defined as SUA ≥360 μmol/L for women and SUA ≥420 μmol/L for men (1, 16). Pregnant or lactating women and patients with cardiovascular diseases, renal diseases, metabolic diseases, tumors, psychiatric diseases, or other diseases were all excluded. Verbal informed consent was obtained from all subjects. All experimental protocols were approved by the Ethics Committee of Binzhou Medical University (No. 2022-352).

### 2.2 Data collection

For laboratory testing, fasting blood was collected from volunteers and sent to hospital laboratories for uniform blood biochemistry testing. The following indicators were measured: UA, alanine aminotransferase (ALT), aspartate aminotransferase (AST), total protein (TP), albumin (ALB), total protein/albumin (A/G), total bilirubin (TBIL), direct bilirubin (DBIL), indirect bilirubin (IBIL), γ-glutamyl transferase (GGT), creatinine (Crea), urea nitrogen (Urea), TGs, high-density lipoprotein cholesterol (HDL-C), low-density lipoprotein cholesterol (LDL-C), and glucose (GLU).

### 2.3 Random forest method

The random forest (RF) method is an ensemble learning algorithm derived from the development of decision trees, which uses randomization to create a large number of decision trees. It is the most recent algorithm that can manage missing and unbalanced data, and it is suitable for analyzing complex data (17). As a combinatorial classifier, the RF method uses the bootstrap resampling method to extract multiple sample sets from the training samples. The RF method uses the extracted sample sets to construct a decision tree model, and it gathers several decision trees together to obtain the final result through majority voting or averaging. Because the RF method has high stability, high prediction accuracy, and is not prone to overfitting, it is widely used in disease prediction. Naveed (18) used the RF algorithm to classify benign and malignant breast lesions with an accuracy of 98%, improving system efficiency, reducing human error, and ultimately developing a method that allows early detection of breast cancer.

### 2.4 Support vector machine

A support vector machine (SVM) is a generalized linear classifier that classifies binary data according to supervised learning. The basic model of a SVM is a classifier that defines the maximum interval in the feature space, which is suitable for small sample data, and SVMs can solve high-dimensional problems. In general, larger spacings indicate greater differences between two sample types, allowing easier distinction between the sample types. Han et al. (19) combined a SVM with proteomics methods to identify biomarkers that predict chemoresistance in small cell lung cancer (SCLC), providing a useful method for the treatment of SCLC. In addition, Dong et al. (20) developed a highly accurate risk prediction model for depression in patients with systemic lupus erythematosus (SLE) using a SVM.

### 2.5 Statistical analysis

In the first stage, all samples were grouped by gender and age, and the risk factors of HUA in each group were analyzed. All data were analyzed using IBM SPSS Statistics 26 and GraphPad Prism 8. Chi-square tests were used to compare categorical variables. For quantitative variables, Mann–Whitney *U* test was used to compare baseline characteristics between groups. The qualitative data were tested by the *χ*^2^ test. Pearson correlation analysis was used to compare the degree of association between SUA and each biochemical index, and a correlation coefficient of |*r*| > 0.1 indicated the presence of correlation. Risk factors for HUA were subsequently analyzed using multifactorial binary logistic regression. Finally, the AUC of the receiver operating characteristic curve (ROC) was used to evaluate the predictive value and practical significance of the biochemical indexes. All statistical analyses were two-sided, and *p* < 0.05 was considered statistically significant.

In the second stage, 15 biochemical indexes were tested by Mann–Whitney *U* test. Additionally, gender and age (young, ≤39 years; middle age, 40–59 years; and older adults, ≥60 years) were training features, and the presence or absence of disease was the target vector. The RF and SVM models were constructed using the e1071 (version 1.7-13, https://CRAN.R-project.org/package=e1071) and RandomForest (version 4.7-1, https://CRAN.R-project.org/doc/Rnews/) R packages. In addition, 70% of the samples was used as the training set to build the model, and the remaining 30% of the samples was used as the teat set for testing and evaluating the model. The presence or absence of HUA was the target vector, and the model was ten-fold cross-verified. The original data set was randomly divided into ten parts as follows: nine parts were used as the training set; and one part was used as the test set. The risk value of HUA onset was calculated for each individual in the validation set and combined with the actual diagnostic results of each individual in the validation set. Finally, the optimal model was selected for feature extraction and AUC evaluation.

## 3 Results

### 3.1 Clinical characteristics of the participants

The present study included 334 females, with an average age of 47 (37, 56) years and an UA level of 334.20 (242.73, 432.88) μmol/L. There were 371 male participants, with an average age of 48 (35, 56) years and an UA level of 428.10 (326.10, 513.70) μmol/L. The UA level in males was significantly higher than females (*p* < 0.05). The samples were divided into three groups based on age, namely, young (≤39 years), middle age (40–59 years), and older adults (≥60 years). The age distribution of the population is shown in Table 1.

**Table 1:** Characteristics of the population with HUA.

Variable	HC	HUA	*χ* ^2^	*p*
Gender			2.928	0.087
Male	176	19 5		
Female	180	154		
Age			23.276	<0.001
Youth	112	119		
Middle age	215	162		
Old age	29	68		

### 3.2 Comparison of biochemical indicators of HUA by gender

For males, the UA, ALT, AST, GGT, Urea, Crea, TG, LDL-C, and GLU levels were significantly higher in the HUA group compared to the HC group, while the TP level was significantly lower in the HUA group compared to the HC group (Table 2). For females, the UA, ALT, AST, TBIL, DBIL, IBIL, GGT, Urea, Crea, TG, LDL-C, and GLU levels were significantly higher in the HUA group than those in the HC group, while the ALB and A/G levels were significantly lower in the HUA group compared to the HC group (*p* < 0.05) (Table 3).

**Table 2:** Comparison of biochemical indicators in males between the HUA and HC groups.

Variable	HUA group (*n*=195)	HC group (*n*=176)	*Z*	*P*
UA (umol/L)	510.1(456.6, 551.8)	319(280.23, 369.45)	−27.371	<0.001
ALT (U/L)	25.4(19.8, 35)	20(15, 26)	−3.937	<0.001
AST (U/L)	23(19, 32.2)	19(16, 22.58)	−3.973	<0.001
TP (g/L)	71.1(61.4, 74.9)	71.3(68.13, 74.48)	6.645	<0.001
GGT (U/L)	37(24, 57)	22(18, 31.8)	−4.989	<0.001
Urea (mmol/L)	5.22(4.46, 6.19)	4.99(4.41, 5.81)	−2.913	0.004
Crea (umol/L)	79.6(72.8, 87.4)	71.7(65.38, 77.53)	−6.547	<0.001
TG (mmol/L)	1.41(1.11, 2.19)	1.29(0.92, 1.69)	−3.232	0.001
LDL-C (mmol/L)	2.63(2.01, 3.32)	2.33(1.72, 2.77)	−3.985	<0.001
GLU (mmol/L)	5.23(4.77, 5.71)	4.99(4.66, 5.50)	−2.444	0.015

**Table 3:** Comparison of biochemical indicators in females between the HUA and HC groups.

Variable	HUA group (*n*=154)	HC group (*n*=180)	*Z*	*P*
UA (umol/L)	440.25(400.78, 479.3)	249.6(197.05, 302.13)	−25.71	<0.001
ALT (U/L)	20.9(16, 31.03)	16(12, 22)	−5.314	<0.001
AST (U/L)	20.25(15, 27.15)	17.65(14, 22)	−3.097	0.002
ALB (g/L)	43.9(38.78, 46.2)	44.6(42.83, 46.58)	4.930	<0.001
A/G	1.4(1.3, 1.6)	1.6(1.5, 1.7)	3.654	<0.001
TBIL (umol/L)	11.52(8.28, 16.35)	10.6(8.33, 13.3)	−2.208	0.029
DBIL (umol/L)	2.77(1.67, 3.9)	11.52(8.28, 16.35)	−2.047	0.042
IBIL (umol/L)	8.56(6.18, 12.35)	8.05(6.1, 10.08)	−2.203	0.029
GGT (U/L)	22(15.93, 32.63)	14.05(12, 18.95)	−3.474	0.001
Urea (mmol/L)	4.85(4.2, 6.79)	4.42(3.8, 5.20)	−4.506	<0.001
Crea (umol/L)	64.05(56, 76.5)	56.1(51.28, 62)	−2.873	0.005
TG (mmol/L)	1.37(1.06, 1.85)	1.33(0.86, 1.66)	−2.789	0.006
LDL-C (mmol/L)	2.41(1.77, 3.36)	2.23(1.49, 2.80)	−3.553	<0.001
GLU (mmol/L)	5.32(4.90, 5.93)	4.87(4.57, 5.13)	−5.483	<0.001

**Figure 1:**
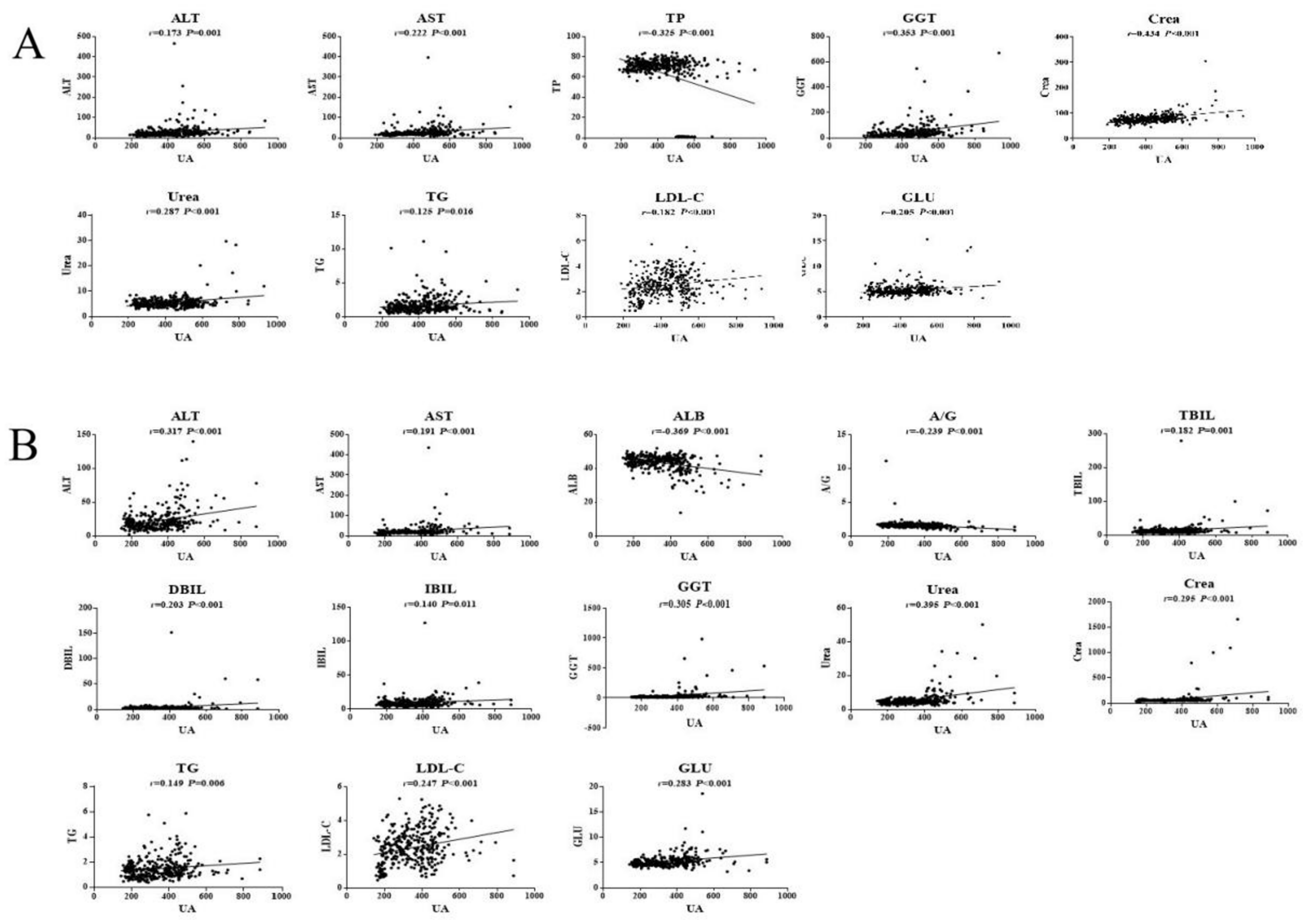
Pearson correlation analysis of HUA in males **(A)** and females **(B)**.

Pearson correlation analysis showed that the ALT, AST, GGT, Urea, Crea, TG, LDL-C, and GLU levels were positively correlated with SUA in males, whereas the TP level was negatively correlated with SUA in males (*p* < 0.05). Among the serum biochemical indicators, TP, GGT and Crea had an |*r*| > 0.3, indicating a good correlation with UA (Figure 1A). Moreover, Pearson correlation analysis demonstrated that the ALT, AST, TBIL, DBIL, IBIL, GGT, Urea, Crea, TG, LDL-C, and GLU levels were significantly positively associated with SUA in females, whereas SUA was negatively associated with the levels of ALB and A/G in females. Among the serum biochemical indicators, ALT, ALB, GGT, and Urea had an |*r*| > 0.3, indicating a good correlation with UA (Figure 1B).

#### 3.2.1 Logistic regression analysis of HUA biochemical indicators according to gender

The biochemical indicators of HUA were further analyzed by multivariate binary logistic regression, considering the presence or absence of a disease as the dependent variable (HUA = 1, HC = 0) and the other indicators as independent variables. The levels of Crea [odds ratio (OR) = 1.091, 95% confidence interval (CI): 1.062–1.121, *p* < 0.001], TG (OR = 1.374, 95% CI: 1.071–1.763, *p* = 0.012), LDL-C (OR = 1.839, 95% CI: 1.362–2.482, *p* < 0.001), and GGT (OR = 1.014, 95% CI: 1.001–1.026, *p* = 0.028) were independent risk factors for HUA in the male population. For every 1 mmol/L increase in LDL-C, the risk significantly increases by 84% (Supplementary Table S1).

In addition, the levels of Crea (OR = 1.084, 95% CI: 1.055–1.115, *p* < 0.001), LDL-C (OR = 1.461, 95% CI: 1.109–1.925, *p* = 0.007), and GLU (OR = 3.225, 95% CI: 2.047–5.081, *p* < 0.001) were risk factors for HUA in the female population. For every 1 mmol/L increase in GLU, the risk significantly increases by 223%, and for every 1 mmol/L increase in LDL-C, the risk increases by 46% (Supplementary Table S2).

#### 3.2.2 Analysis of the predictive value of HUA biochemical markers according to gender

**Figure 2:**
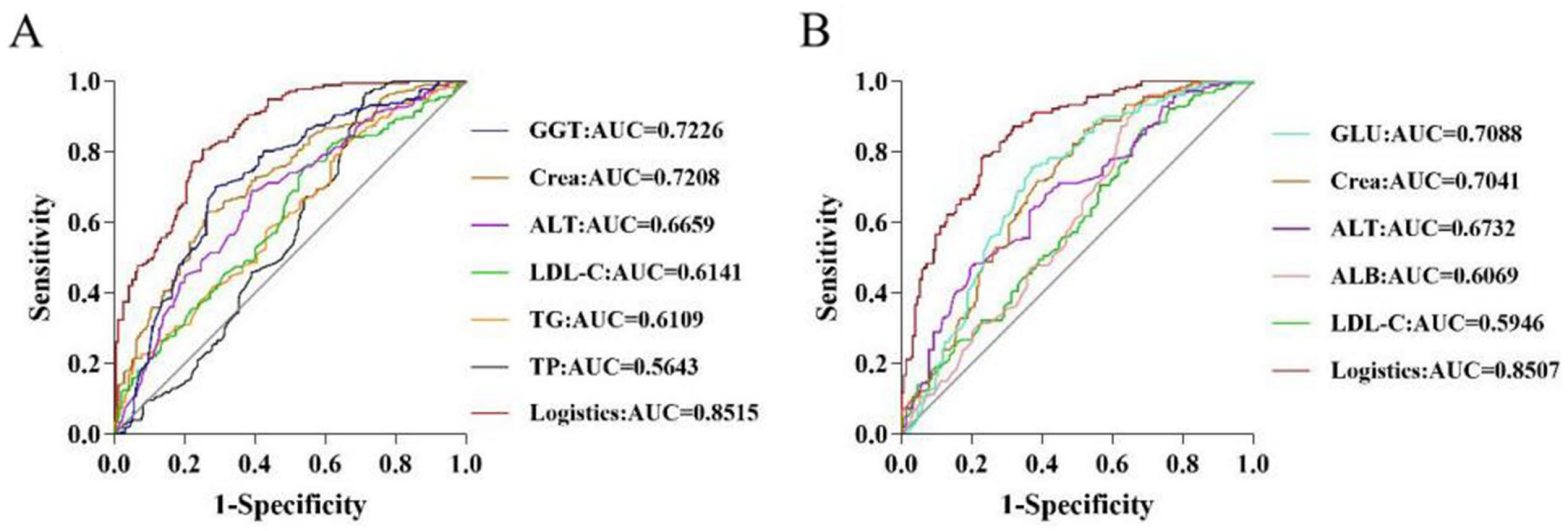
ROC analysis of potential biomarkers in males **(A)** and females **(B)**.

To assess the value of a single biochemical indicator on HUA, ROC curves were plotted, and AUC values were calculated. For males, analysis of the predictive value of a single differential indicator showed that the AUC values of GGT and Crea were 0.7226 and 0.7208, respectively, indicating high predictive value (AUC >0.7). However, the predictive power of individual biochemical indicators was limited. Therefore, the present study established a prediction model based on logistic regression analysis to further improve the performance, sensitivity, and specificity of the prediction model. The logistic factor (AUC = 0.8515, sensitivity = 0.749, and specificity = 0.783) obtained by logistic regression provided a better prediction compared to the single individual indicator (Supplementary Table S3 and Figure 2A). For females, analysis of the predictive value of a single differential indicator demonstrated that the AUC values of GLU and Crea were 0.7088 and 0.7041, respectively, indicating high predictive value (AUC >0.7). The logistic factor (AUC = 0.8507, sensitivity = 0.773, specificity = 0.783) obtained by logistic regression provided a better prediction by including the combined risk factors for HUA (Supplementary Table S4 and Figure 2B).

### 3.3 Comparison of HUA biochemical indicators by age

Cui L et al. reported that SUA levels and the prevalence of HUA are not linearly related to age but rather are related in a “U” or “J” manner, which may be due to the lifestyle of various age groups (21). Therefore, the participants were divided into three groups according to age to identify risk factors and potential biomarkers based on age.

**Figure 3:**
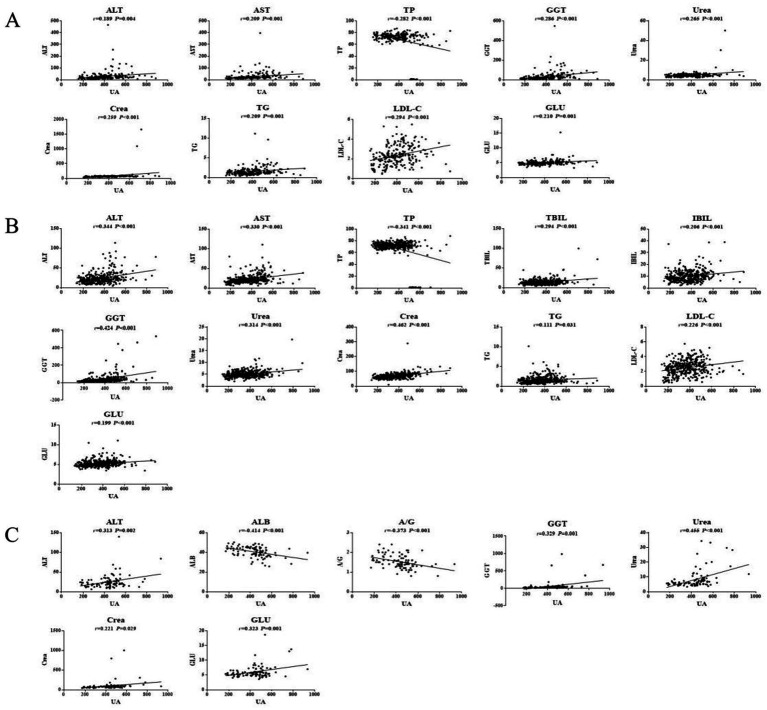
Pearson correlation analysis of HUA in the young **(A)**, middle age **(B)**, and older adults **(C)** groups.

In young participants, the UA, ALT, AST, GGT, Urea, Crea, TG, LDL-C, and GLU levels in the HUA group were significantly higher than those in the HC group, while the TP and A/G levels were significantly lower in the HUA group compared to the HC group (*p* < 0.05) (Table 4). Pearson correlation analysis indicated that the ALT, AST, GGT, Urea, Crea, TG, LDL-C, and GLU levels were positively related to SUA, whereas TP and A/G were negatively related to SUA (*p* < 0.05) (Figure 3A).

**Table 4:** Comparison of the HUA and HC groups in young participants.

Variable	HUA group (*n*=119)	HC group (*n*=112)	*Z*	*P*
UA (umol/L)	474(435.2, 547.2)	296.8(243.5, 329.78)	−19.552	<0.001
ALT (U/L)	24.1(17.3, 34)	16(12, 22.8)	−3.437	0.001
AST (U/L)	21(17, 36)	18(14.05, 21.75)	−3.287	0.001
TP (g/L)	73.5(67.3, 77)	73.05(70.1, 75.88)	3.819	<0.001
A/G	1.62(1.4, 1.8)	1.7(1.56, 1.90)	2.043	0.042
GGT (U/L)	29(18, 49.6)	17.4(13, 24.75)	−4.315	<0.001
Urea (mmol/L)	4.68(4, 5.63)	4.43(3.6, 5.12)	−2.175	0.031
Crea (umol/L)	73.5(63.2, 82.4)	62.6(55.1, 73.3)	−1.993	0.049
TG (mmol/L)	1.33(1.05, 2.01)	1.24(0.91, 1.54)	−3.485	0.001
LDL-C (mmol/L)	2.44(1.87, 3.35)	2.09(1.34, 2.51)	−4.562	<0.001
GLU (mmol/L)	5.02(4.71, 5.37)	4.845(4.45, 5.05)	−3.207	0.002

In middle-aged participants, the UA, ALT, AST, TBIL, IBIL, GGT, Urea, Crea, TG, LDL-C, and GLU levels in the HUA group were significantly higher compared to the HC group, while the TP level was significantly lower in the HUA group compared to the HC group (*p* < 0.05). Pearson correlation analysis showed that the ALT, AST, TBIL, IBIL, GGT, Urea, Crea, TG, LDL-C, and GLU levels were positively associated with SUA, whereas the TP levels were negatively associated with SUA (*p* < 0.05). Among the biochemical indicators, ALT, AST, TP, GGT, Urea, and Crea had an |*r*| > 0.3, indicating a good correlation with UA (Table 5 and Figure 3B).

**Table 5:** Comparison of the HUA and HC groups in middle-aged participants.

Variable	HUA group (*n*=162)	HC group (*n*=215)	*Z*	*P*
UA (umol/L)	460.8(426.75, 518.68)	287.1(233.6, 337.6)	−25.183	<0.001
ALT (U/L)	23(17.9, 34.13)	18.4(14, 24.5)	−5.417	<0.001
AST (U/L)	22(18, 30.2)	18(15, 22)	−5.237	<0.001
TP (g/L)	71.9(67.73, 76.35)	72.2(69, 74.7)	4.533	<0.001
TBIL (umol/L)	12.85(9.9, 17)	11.9(9.3, 15.6)	−2.332	0.021
IBIL (umol/L)	9.78(7.38, 12.74)	8.8(6.5, 12.1)	−2.314	0.021
GGT (U/L)	30.8(19, 51.95)	18.5(14, 25.7)	−4.599	<0.001
Urea (mmol/L)	4.97(4.36, 6.01)	4.7(4.12, 5.6)	−3.121	0.002
Crea (umol/L)	73.85(63.78, 84.3)	63(55.1, 72)	−6.114	<0.001
TG (mmol/L)	1.42(1.14, 2.05)	1.32(0.87, 1.75)	−2.399	0.017
LDL-C (mmol/L)	2.67(1.95, 3.24)	2.37(1.71, 2.92)	−3.150	0.002
GLU (mmol/L)	5.33(4.93, 5.77)	4.95(4.66, 5.33)	−4.112	<0.001

In older adults participants, the UA, ALT, AST, GGT, Urea, Crea, and GLU levels in the HUA group were significantly higher than those in the HC group, whereas the levels of ALB and A/G were significantly lower in the HUA group compared to the HC group (*p* < 0.05). Pearson correlation analysis showed that the ALT, GGT, Urea, Crea, TG, and GLU levels were positively correlated with SUA, while the levels of ALB and A/G were negatively correlated with SUA (*p* < 0.05). Among the biochemical indicators, ALT, ALB, A/G, GGT, Urea, and GLU had an |*r*| > 0.3, indicating a good correlation with UA (Table 6 and Figure 3C).

**Table 6:** Comparison of the HUA and HC groups in older adults participants.

Variable	HUA group (*n*=68)	HC group (*n*=29)	*Z*	*P*
UA (umol/L)	487.5(446.68, 536.85)	277(230.9, 316.5)	−12.172	<0.001
ALT (U/L)	21.75(17.63, 32.83)	18.2(13.85, 23.3)	−3.170	0.002
AST (U/L)	21.2(15.23, 29.25)	20.9(16, 24.8)	−2.037	0.045
ALB (g/L)	39.55(36.55, 43.7)	44.2(41.1, 46.05)	3.733	<0.001
A/G	1.4(1.27, 1.59)	1.68(1.5, 2)	4.909	<0.001
GGT (U/L)	29(20.43, 43.45)	19.4(14, 29.5)	−2.473	0.016
Urea (mmol/L)	6.99(5.32, 10.35)	5.5(4.61, 6.11)	−4.528	0.012
Crea (umol/L)	80.75(66.15, 96)	68.6(55.15, 76.05)	−2.591	0.012
GLU (mmol/L)	5.67(4.87, 7.11)	5.33(4.91, 5.81)	−2.903	0.005

#### 3.3.1 Logistic regression analysis of HUA biochemical indicators according to age

The risk factors for HUA were further identified by multifactorial logistic regression. LDL-C (OR = 1.949, 95% CI: 1.401–2.712, *p* < 0.001), GLU (OR = 1.941, 95% CI: 1.136–3.314, *p* = 0.015), TG (OR = 1.750, 95% CI: 1.071–2.859, *p* = 0.026), and Crea (OR = 1.044, 95% CI: 1.018–1.070, *p* = 0.001) were independent risk factors for HUA in young participants (Supplementary Table S5). For every 1 mmol/L increase in LDL-C, the risk significantly increases by 95%, and for every 1 mmol/L increase in TGs, the risk increases to 75%.

In addition, GLU (OR = 1.583, 95% CI: 1.178–2.128, *p* = 0.002), LDL-C (OR = 1.445, 95% CI: 1.121–1.862, *p* = 0.004), and Crea (OR = 1.052, 95% CI: 1.033–1.073, *p* = 0.000) were risk factors for HUA in middle-aged participants (Supplementary Table S6). In older adults participants, GLU (OR = 1.781, 95% CI: 1.045–3.034, *p* = 0.034), ALT (OR = 1.074, 95% CI: 1.009–1.144, *p* = 0.026), and Crea (OR = 1.055, 95% CI: 1.018–1.093, *p* = 0.003) were risk factors for HUA (Supplementary Table S7).

#### 3.3.2 Analysis of the predictive value of HUA biochemical markers according to age

**Figure 4:**
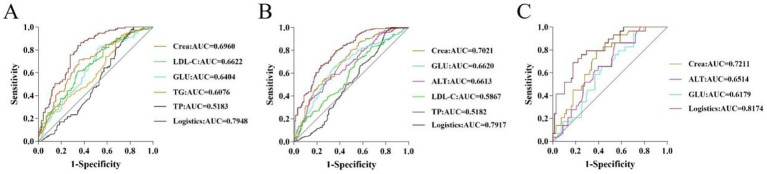
ROC curves of potential biomarkers in young **(A)**, middle-aged **(B)**, and older adults **(C)** participants.

To evaluate the diagnostic efficacy of clinical indicators on diseases, an ROC curve was plotted with the false positive rate (1-specificity) as the abscissa and the true positive rate (sensitivity) as the ordinate. For young participants, Crea and LDL-C had great predictive power (AUC >0.65), and the logistic factor (AUC = 0.7948, sensitivity = 0.664, specificity = 0.839) was significantly better than the individual biochemical indicators (Supplementary Table S8 and Figure 4A). In addition, the AUC values of Crea, GLU, and ALT were 0.7021, 0.6620, and 0.6613, respectively, for middle-aged individuals, and the AUC values of Crea and ALT were 0.7211 and 0.6514, respectively, for older adults individuals. The logistic predictive values for middle-aged and older adults participants were 0.7917 and 0.8174, which were significantly better than the individual biochemical indicators (Supplementary Tables S9, S10 and Figures 4B,C).

### 3.4 Machine learning

**Figure 5:**
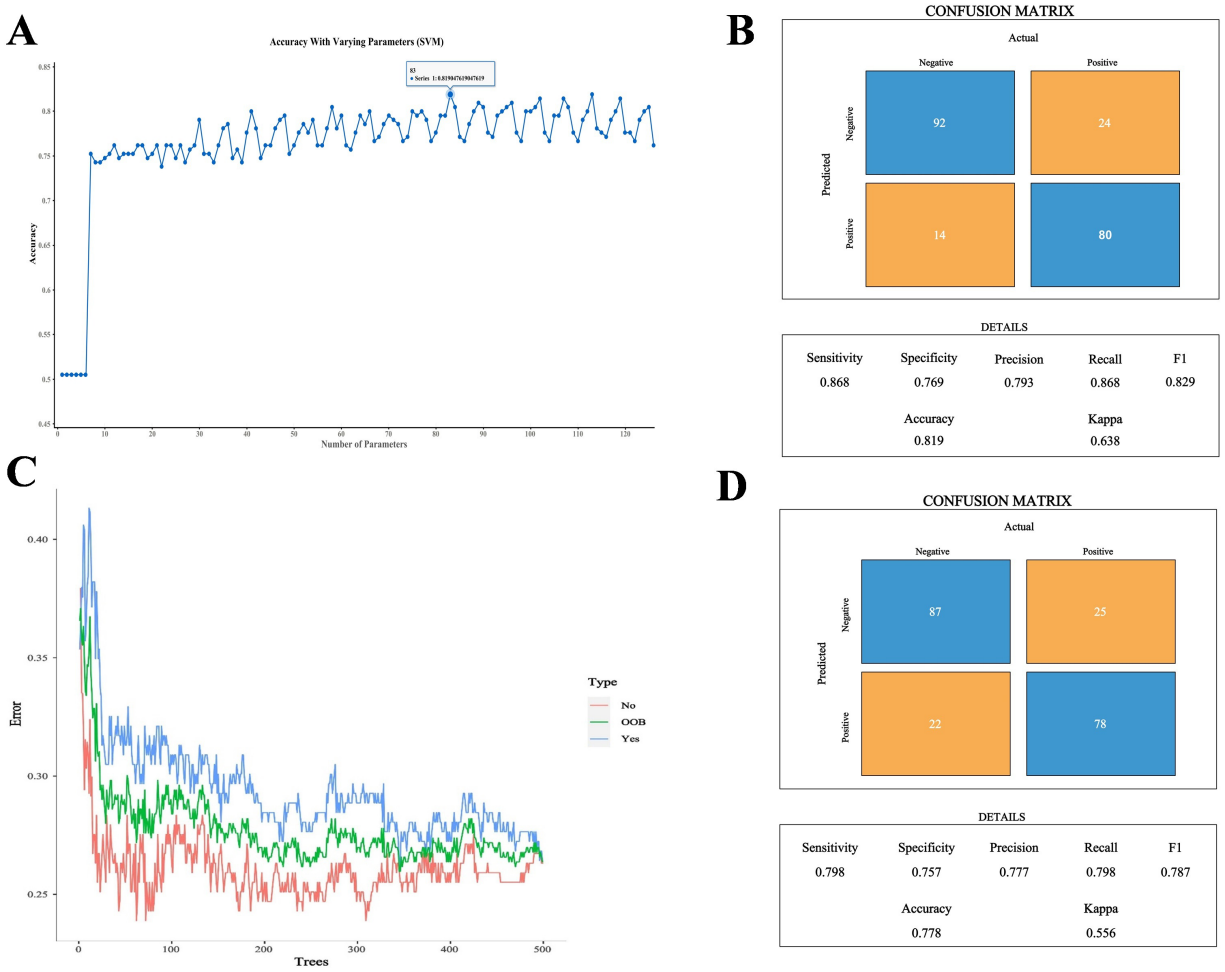
**(A)** Comparison of SVM cross-validation parameters. **(B)** Performance indicators of the optimal SVM parameter model. **(C)** Number of optimal decision trees in the RF method. **(D)** Performance indicators of the optimal RF parameter model.

**Figure 6:**
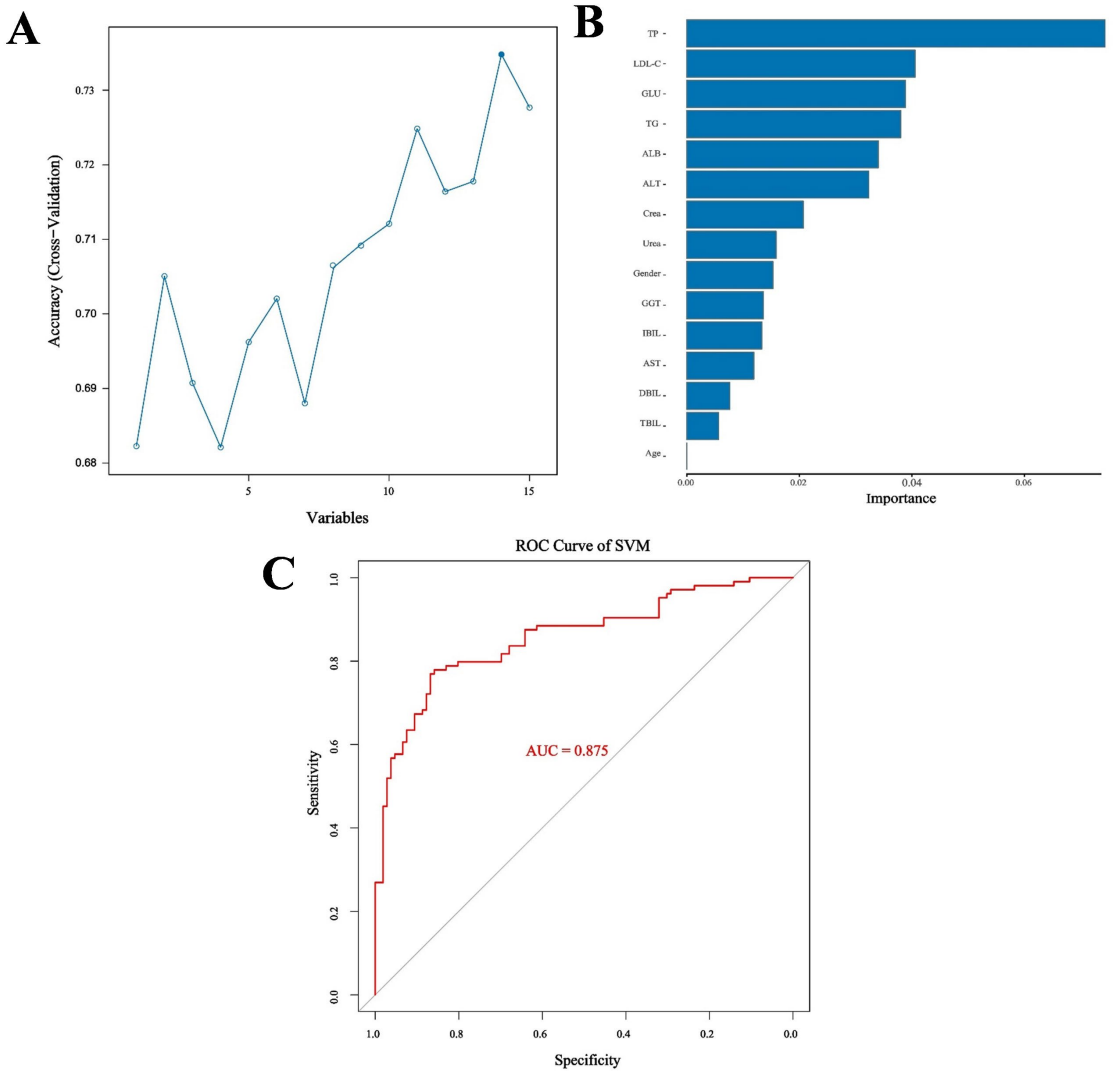
**(A)** Optimal number of features. **(B)** Feature importance ranking. **(C)** ROC curve for SVM.

Because two indicators (A/G and HDL-C) did not meet the requirements for the SVM model according to the Mann–Whitney *U* test, the remaining 13 biochemical indicators, gender, and age were used as the training characteristics (Table 7). The SVM model was tuned using the gamma index and cost index, and 126 parameters were set for ten-fold cross-validation (SVM/SVM_params.csv). The final cross-validation results showed that the 83rd parameter combination had the best effect in constructing the model, corresponding to a cost index of 16 and a gamma index of 0.065. The model was then used to predict the performance indicators of the test set (Figures 5A,B). Moreover, the RF method and out-of-bag (OOB) error were used to select the appropriate number of decision trees, and the test set was utilized for predictive evaluation (Figures 5C,D). For the SVM model, the sensitivity, specificity, precision, recall, F1, accuracy, and kappa value were 0.868, 0.769, 0.793, 0.868, 0.829, 0.819, and 0.638, respectively, all of which were better than the values obtained with the RF method. Accuracy values >0.8 and kappa values >0.6 indicate models with good accuracy and consistency. The SVM model was then used for feature extraction. The model had the best performance when selecting 14 features, except age. The test set was then evaluated and ROC curves were plotted, yielding an AUC value of 0.958 for the training set and 0.875 for the validation set (Figure 6; Supplementary Figure S1).

**Table 7:** Comparison of basic characteristics in the HUA and HC groups.

Variable	HUA group (*n*=349)	HC group (*n*=356)	*Z*	*P*
UA (umol/L)	469(432.4, 531.1)	288.3(235.98, 333.2)	−34.492	<0.001
ALT (U/L)	23.3(17.55, 33.3)	18(13, 24)	−5.896	<0.001
AST (U/L)	21.5(17, 31)	18(15, 22)	−5.098	<0.001
TP (g/L)	71.5(65.6, 76.2)	72.35(69.3, 75.05)	6.398	<0.001
ALB (g/L)	45.2(41.4, 47.5)	45.5(43.2, 47)	3.997	<0.001
TBIL (umol/L)	13(9.53, 17.30)	11.65(9.2, 15.5)	−2.773	0.006
DBIL (umol/L)	3(2.1, 4.5)	2.9(2.1, 4)	−2.348	0.019
IBIL (umol/L)	9.7(6.95, 13.07)	8.65(6.41, 11.65)	−2.826	0.005
GGT (U/L)	29(19, 48)	18(14, 25)	−5.969	<0.001
Urea (mmol/L)	5.1(4.34, 6.26)	4.69(4.08, 5.55)	−5.490	<0.001
Crea (umol/L)	74.6(64.3, 85.25)	63.15(55.1, 72.55)	−3.984	<0.001
TG (mmol/L)	1.39(1.07, 2.05)	1.31(0.88, 1.68)	−4.331	<0.001
LDL-C (mmol/L)	2.5(1.9, 3.32)	2.27(1.65, 2.78)	−5.458	<0.001
GLU (mmol/L)	5.28(4.82, 5.77)	4.92(4.63, 5.3)	−5.769	<0.001

## 4 Discussion

UA is the end product of purine nucleotide catabolism, and as UA levels increase, the risk of lipid metabolism disorders, coronary heart disease, hypertension, T2DM, and obesity gradually increases (22). In recent years, the research focus on HUA has increased. In clinical practice, SUA levels are still used to diagnose HUA, but the goal to control of HUA is not fully defined. Early identification of asymptomatic individuals at high risk of HUA is crucial to help the early prevention and control of HUA. Therefore, the present study developed a new diagnostic model involving routine clinical biochemical markers, and the risk factors for different gender and age groups were analyzed.

### 4.1 Relationship between HUA and liver function indicators

As the largest organ in the human body, the liver has several functions, including nutrient metabolism, bile secretion, immune defense, and blood volume regulation. Hepatocyte cell culture studies have shown that UA can cause mitochondrial oxidative stress, which may lead to liver dysfunction. UA catalyzes an increase in liver inflammation by inducing inflammation and oxidative stress, either directly or through lipids and glucose, leading to hepatocyte death and functional hemocytopenia. The damaged liver further causes the levels of ALT and AST to increase, and the ability to synthesize ALB decreases. In turn, hepatic dysfunction can directly lead to elevated UA levels through multiple mechanisms (23). Chen et al. (24) reported that SUA concentration is positively correlated with ALT levels and that elevated UA levels may be associated with ALT elevation. In the present study, ALT and AST levels were significantly elevated in patients with HUA, which may indicate liver or mitochondrial damage. In particular, Pearson correlation and logistic regression analyses revealed that the ALT levels in middle-aged participants (*r* = 0.344, *p* < 0.001, OR = 1.044) and in older adults participants (*r* = 0.313, *p* = 0.002, OR = 1.074) were both strongly correlated with UA. Nevertheless, there were differences in the distribution of ALT and AST in hepatocytes. ALT is mainly distributed in the hepatocyte, reflecting hepatocyte membrane damage; AST is mainly distributed in hepatocyte mitochondria, and its elevation indicates hepatocyte damage at the organelle level (24, 25). In middle-aged and older adults individuals, AST/ALT >1 indicated that hepatocytes and mitochondria were severely destroyed, and the risk of cirrhosis was increased. Therefore, compared to AST, ALT is a better predictor of HUA, which can detect problems at an early stage of liver damage and control the onset and progression of HUA early.

In the present study, 14 metrics were selected for modeling, including ALT, AST, TP, ALB, TBIL, DBIL, IBIL, GGT, Urea, Crea, TG, LDL-C, GLU, and age. Among them, TP was considered the best indicator for predicting HUA. ALB, which is the main component of TP, was also ranked highly. Han et al. (26) reported that TP is an independent risk factor for HUA in individuals over 80 years of age. In patients with T2DM, ALB was included in the risk prediction model for HUA and was considered an independent risk factor for HUA (27). Compared to the HC group, the TP and ALB levels were significantly reduced in the HUA group. There is evidence of an important correlation between HUA and nonalcoholic fatty liver disease (NAFLD), and high levels of UA may damage liver parenchymal cells (28). The liver, as the only organ that synthesizes albumin, has a strong compensatory function. If there is slight damage to the liver in the early stages of the disease, TP and ALB may not necessarily be significantly altered. Only when the body has chronic liver parenchymal injury, such as chronic hepatitis and cirrhosis, the serum TP and ALB are reduced due to the obvious impairment of hepatocyte protein synthesis function. In addition, SUA causes endothelial dysfunction and low-grade inflammation by inhibiting the production of nitric oxide and reactive oxygen species (ROS). ALB is a negative acute phase-reactive protein, and ALB levels decrease during chronic inflammation. Moreover, HUA is a risk factor for chronic kidney disease (CKD). Studies have shown that the ALB gene is a differentially expressed gene (DEG) for CKD in patients with asymptomatic HUA, and ALB may be a major target of oxidative stress. Therefore, control of SUA is important to reduce complications and delay the progression of kidney disease (29).

As an essential enzyme for glutathione metabolism, GGT can produce free radicals and trigger oxidative stress, indicating that GGT is an independent marker of oxidative stress. In addition, GGT has been associated with the development of hypertension, coronary heart disease, T2DM, and metabolic disorders. By regulating the interaction between leukotriene C4 and leukotriene D4, GGT plays a non-negligible role in inflammation (30, 31). Additionally, Ryoo JH et al. reported that GGT level may be a predictor of the development of insulin resistance (32). A high concentration of insulin can promote renal tubular hydrogen and sodium exchange, increase UA reabsorption, and inhibit UA excretion. Moreover, a high concentration of insulin can also cause an increase in purine metabolism by activating the hexose monophosphate shunt, resulting in an increase in SUA levels (30). In the male population, GGT (*r* = 0.353, *p* < 0.001) was significantly associated with SUA. As a risk factor for HUA, GGT (AUC = 0.7226) was also an independent predictor of disease development. High levels of SUA can induce oxidative stress, which leads to cell damage, resulting in various kidney diseases. Furthermore, GGT is a non-specific indicator of liver damage and may be elevated in patients with hepatobiliary disease or individuals who have high alcohol intake (33). Consistent with the present results, He et al. (34) reported that heavy alcohol consumption in men, but not in women, is associated with the risk of HUA, which may be due to the higher tendency of men to drink alcohol than women.

### 4.2 Relationship between HUA and kidney function indicators

Studies have shown that the presence of HUA is an essential biomarker of renal risk (35). UA crystals are deposited in renal collecting ducts, leading to acute kidney injury and kidney disease. At the same time, high levels of UA can induce oxidative stress and ROS production in vascular endothelial cells, as well as promote the expression of the interleukin (IL)-6 and tumor necrosis factor (TNF)-α pro-inflammatory cytokines (36). Through a cross-sectional study, Lyu et al. (37) reported that Crea is a risk factor for HUA. The present study further demonstrated that Crea levels were significantly higher in the HUA group than in the HC group, indicating potential kidney damage (*p* < 0.05). In addition, Crea had a good predictive value in different populations (AUC >0.65), and the predictive value was higher in the male population (AUC = 0.7208) and older adults participants (AUC = 0.7211). Serum Crea is the most commonly used indicator of renal function, and abnormal UA levels can indirectly affect renal function by affecting serum Crea levels. Due to renal arteriosclerosis and insufficient blood circulation in the kidney, older adults individuals experience a decrease in glomerular filtration rate and renal tubular excretion, eventually resulting in reduced UA excretion and increased blood UA levels (37). Therefore, older adults individuals should be aware of HUA nephropathy.

### 4.3 Relationship between HUA and blood lipids

Dyslipidemia is one of the most common chronic diseases, and it increases the risk of HUA, cardiovascular disease, stroke, and T2DM (38, 39). TGs are esterified from three molecules of fatty acids and one molecule of glycerol, and they are the most abundant lipids in the human body. Multivariate logistic regression showed that TG (OR = 1.374, 95% CI: 1.071–1.763, *p* = 0.012) and LDL-C (OR = 1.839, 95% CI: 1.362–2.482, *p* < 0.001) levels were risk factors for HUA in men. For every 1 mmol/L increase in TGs and LDL-C, the risk increases by 37 and 84%, respectively. Fatty acid synthesis is related to *de novo* purine synthesis, and when the content of TGs increases, it promotes the production and utilization of free fatty acids, thereby accelerating the production of UA (40). In the present study, LDL-C and TGs ranked second and fourth in feature extraction, respectively, suggesting that they play a role in the prediction of HUA. Moreover, LDL-C and TG levels were significantly increased in the HUA group. Increased lipoprotein may lead to decreased SUA clearance. Dyslipidemia is common in HUA patients, and elevated levels of TG and TC are considered independent risk factors for HUA (29). Ye et al. (41) reported that higher UA levels are associated with an increased risk of developing LDL-C abnormalities in children, and they suggested that there is a 2.9% increased risk of high LDL-C with a 10 μmol/L increase in UA levels. As humans age, TG clearance decreases, plasma TG increases and continuous deposition of visceral adipose tissue increases, all of which indicate an elevated risk of metabolic disease (8). Therefore, men, especially middle-aged and older adults men, are considered a high-risk group for HUA and should pay special attention to daily dietary habits and regular physical examinations for early prevention and disease control.

### 4.4 Relationship between HUA and blood GLU

In the present study, the prevalence of HUA in the entire population was 16.20%, while the prevalence of HUA in individuals with T2DM was 17.24% (42). Guo and Xu (43) reported that insulin resistance is positively correlated with SUA in women (*p* < 0.05) and that insulin sensitivity is lower in postmenopausal women than in premenopausal women. Insulin resistance can cause blood glucose metabolism disorders and increase liver fat synthesis, and it can eventually lead to purine metabolism disorders, thus raising blood UA levels. Liu et al. (44) demonstrated that the risk factor for HUA in women is high blood GLU (OR = 1.508; 95% CI: 1.084–2.099), and the risk increases by 50.8% for every 1 mmol increase in blood GLU. Consistently, the present study demonstrated that GLU (OR = 3.225; 95% CI: 2.047–5.081; AUC = 0.7088) was a risk factor for HUA in women, with good predictive value. Logistic regression analysis and ROC curves indicated that serum GLU was a common risk factor in the three age groups, namely, young, middle age, and older adults, and the cut-off value increased with age. In addition, GLU also performed well with the HUA model predictions. The prevalence of uric acidemia increases with age, and the risk point for women is 50 years. Because most women after the age of 50 enter menopause, estrogen secretion decreases, and its protective effect on the body gradually declines (45). Therefore, individuals, especially middle-aged women, should pay close attention to their blood sugar changes during physical examinations and take active measures to correct poor dietary habits to strictly control the intake of purines and foods with high levels of sugar or oil.

The present cross-sectional survey had certain limitations. For example, the causal relationship between the screened biochemical indexes and UA could not be confirmed, and the dynamic changes of biochemical indicators could not be captured. Although there were exclusion criteria for the included population, confounding factors, such as work style and education status, were not included in the scope of correction.

## 5 Conclusion

The present study analyzed the risk factors for HUA according to different genders and ages using easily available physical examination indicators to establish a simple prediction model for HUA. TP, LDL-C, and GLU levels are risk factors important for predicting HUA. The present study identified high-risk groups and screened out clinically meaningful indicators in different groups. However, the influencing factors of HUA are complex and diverse, and additional mechanistic studies are needed to verify these factors to promote the prevention and treatment of HUA.

## Data Availability

The raw data supporting the conclusions of this article will be made available by the authors, without undue reservation.
