# Supplementary figures and images for: Study on risk factor analysis and model prediction of hyperuricemia in different populations
Source: Front Nutr. 2024 Oct 14;11:1417209. doi: 10.3389/fnut.2024.1417209 (PMC11513274; doi:10.3389/fnut.2024.1417209)

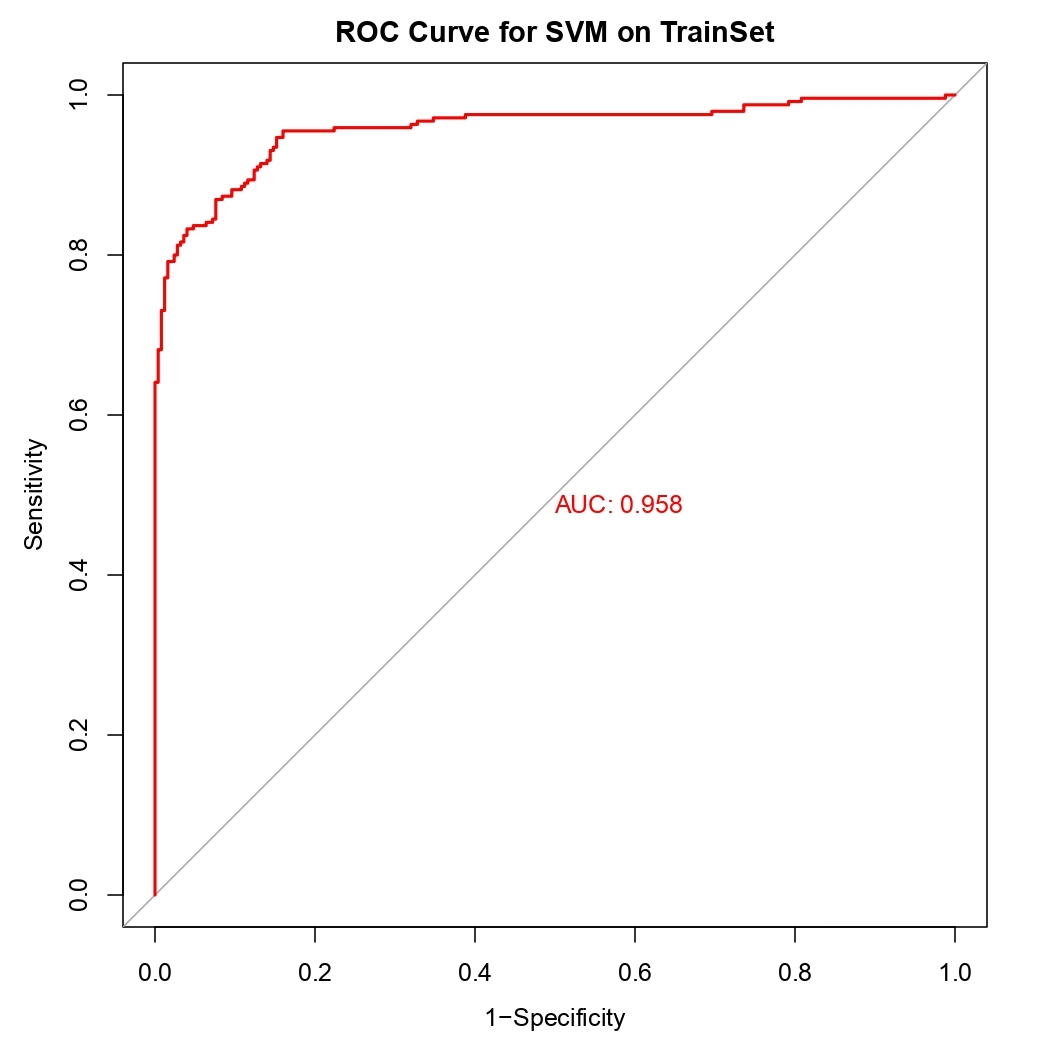

Supplement: Supplementary file 1 [file Image_1.JPEG]
