# Supplementary material for: Study on risk factor analysis and model prediction of hyperuricemia in different populations
Source: Front Nutr. 2024 Oct 14;11:1417209. doi: 10.3389/fnut.2024.1417209 (PMC11513274; doi:10.3389/fnut.2024.1417209)
Supplement: Supplementary file 2 [file Table_1.DOCX]

Supplementary Table 1 Multivariate logistic regression analysis in males.

| Variable | *B* | *SE* | *wald* | *P* | *OR* | *95%CI* | |
| --- | --- | --- | --- | --- | --- | --- | --- |
|  |  |  |  |  |  | *Lower limit* | *Upper limit* |
| ALT | 0.023 | 0.010 | 5.110 | 0.024 | 1.023 | 1.003 | 1.043 |
| TP | -0.053 | 0.013 | 16.785 | <0.001 | 0.948 | 0.924 | 0.973 |
| GGT | 0.014 | 0.006 | 4.802 | 0.028 | 1.014 | 1.001 | 1.026 |
| Crea | 0.088 | 0.014 | 40.125 | <0.001 | 1.091 | 1.062 | 1.121 |
| TG | 0.318 | 0.127 | 6.262 | 0.012 | 1.374 | 1.071 | 1.763 |
| LDL-C | 0.609 | 0.153 | 15.819 | <0.001 | 1.839 | 1.362 | 2.482 |

Supplementary Table 2 Multivariate logistic regression analysis in females.

| Variable | *B* | *SE* | *wald* | *P* | *OR* | *95%CI* | |
| --- | --- | --- | --- | --- | --- | --- | --- |
|  |  |  |  |  |  | *Lower limit* | *Upper limit* |
| ALT | 0.052 | 0.013 | 15.083 | <0.001 | 1.054 | 1.026 | 1.082 |
| ALB | -0.082 | 0.038 | 4.683 | 0.030 | 0.921 | 0.855 | 0.992 |
| Crea | 0.081 | 0.014 | 32.519 | <0.001 | 1.084 | 1.055 | 1.115 |
| LDL-C | 0.379 | 0.141 | 7.263 | 0.007 | 1.461 | 1.109 | 1.925 |
| GLU | 1.171 | 0.232 | 25.479 | <0.001 | 3.225 | 2.047 | 5.081 |

Supplementary Table 3 AUC and efficacy assessment of biochemical markers and combined factors in males.

| Variable | Cut-off value | Sensitivity | Specificity | *P* | AUC |
| --- | --- | --- | --- | --- | --- |
| GGT | 27.90 | 0.713 | 0.699 | <0.0001 | 0.7226 |
| Crea | 77.00 | 0.626 | 0.744 | <0.0001 | 0.7208 |
| ALT | 23.25 | 0.610 | 0.687 | <0.0001 | 0.6659 |
| LDL-C | 2.825 | 0.462 | 0.761 | 0.0001 | 0.6141 |
| TG | 1.355 | 0.564 | 0.585 | 0.0002 | 0.6109 |
| TP | 64.05 | 0.287 | 0.966 | 0.0322 | 0.5643 |
| Logistics | 0.5061384 | 0.749 | 0.807 | <0.0001 | 0.8515 |

Supplementary Table 4 AUC and efficacy assessment of biochemical markers and combined factors in females.

| Variable | Cut-off value | Sensitivity | Specificity | *P* | AUC |
| --- | --- | --- | --- | --- | --- |
| GLU | 5.135 | 0.630 | 0.756 | <0.0001 | 0.7088 |
| Crea | 60.50 | 0.617 | 0.711 | <0.0001 | 0.7041 |
| ALT | 18.45 | 0.636 | 0.633 | <0.0001 | 0.6732 |
| ALB | 44.75 | 0.422 | 0.517 | 0.0008 | 0.6069 |
| LDL-C | 2.685 | 0.435 | 0.706 | 0.0029 | 0.5946 |
| Logistics | 0.4297428 | 0.773 | 0.783 | <0.0001 | 0.8507 |

Supplementary Table 5 Multivariate logistic regression analysis in young participants.

| Variable | *B* | *SE* | *wald* | *P* | *OR* | *95%CI* | |
| --- | --- | --- | --- | --- | --- | --- | --- |
|  |  |  |  |  |  | *Lower limit* | *Upper limit* |
| TP | -0.03 | 0.013 | 5.517 | 0.019 | 0.971 | 0.947 | 0.995 |
| Crea | 0.043 | 0.013 | 11.325 | 0.001 | 1.044 | 1.018 | 1.070 |
| TG | 0.56 | 0.251 | 4.989 | 0.026 | 1.750 | 1.071 | 2.859 |
| LDL-C | 0.667 | 0.169 | 15.672 | <0.001 | 1.949 | 1.401 | 2.712 |
| GLU | 0.663 | 0.273 | 5.893 | 0.015 | 1.941 | 1.136 | 3.314 |

Supplementary Table 6 Multivariate logistic regression analysis in middle-aged participants.

| Variable | *B* | *SE* | *wald* | *P* | *OR* | *95%CI* | |
| --- | --- | --- | --- | --- | --- | --- | --- |
|  |  |  |  |  |  | *Lower limit* | *Upper limit* |
| ALT | 0.043 | 0.011 | 15.802 | <0.001 | 1.044 | 1.022 | 1.066 |
| TP | -0.033 | 0.012 | 8.263 | 0.004 | 0.967 | 0.946 | 0.990 |
| Crea | 0.051 | 0.010 | 27.685 | <0.001 | 1.052 | 1.033 | 1.073 |
| LDL-C | 0.368 | 0.130 | 8.073 | 0.004 | 1.445 | 1.121 | 1.862 |
| GLU | 0.459 | 0.151 | 9.267 | 0.002 | 1.583 | 1.178 | 2.128 |

Supplementary Table 7 Multivariate logistic regression analysis in elderly participants.

| Variable | *B* | *SE* | *wald* | *P* | *OR* | *95%CI* | |
| --- | --- | --- | --- | --- | --- | --- | --- |
|  |  |  |  |  |  | *Lower limit* | *Upper limit* |
| ALT | 0.071 | 0.032 | 4.98 | 0.026 | 1.074 | 1.009 | 1.144 |
| Crea | 0.054 | 0.018 | 8.861 | 0.003 | 1.055 | 1.018 | 1.093 |
| GLU | 0.577 | 0.272 | 4.508 | 0.034 | 1.781 | 1.045 | 3.034 |

Supplementary Table 8 AUC and efficacy assessment of biochemical markers and combined factors in young participants.

| Variable | Cut-off value | Sensitivity | Specificity | *P* | AUC |
| --- | --- | --- | --- | --- | --- |
| Crea | 69.350 | 0.639 | 0.714 | <0.0001 | 0.6960 |
| LDL-C | 2.210 | 0.664 | 0.607 | <0.0001 | 0.6622 |
| GLU | 5.075 | 0.487 | 0.804 | 0.0002 | 0.6404 |
| TG | 1.495 | 0.412 | 0.741 | 0.0047 | 0.6076 |
| TP | 71.850 | 0.429 | 0.643 | 0.6314 | 0.5183 |
| Logistics | 0.5447663 | 0.664 | 0.839 | <0.0001 | 0.7948 |

Supplementary Table 9 AUC and efficacy assessment of biochemical markers and combined factors in middle-aged participants.

| Variable | Cut-off value | Sensitivity | Specificity | *P* | AUC |
| --- | --- | --- | --- | --- | --- |
| Crea | 69.800 | 0.593 | 0.698 | <0.0001 | 0.7021 |
| GLU | 5.155 | 0.648 | 0.656 | <0.0001 | 0.6620 |
| ALT | 20.100 | 0.667 | 0.591 | <0.0001 | 0.6613 |
| LDL-C | 2.625 | 0.519 | 0.623 | 0.0039 | 0.5867 |
| TP | 70.550 | 0.426 | 0.656 | 0.5456 | 0.5182 |
| Logistics | 0.3490111 | 0.765 | 0.665 | <0.0001 | 0.7917 |

Supplementary Table 10 AUC and efficacy assessment of biochemical markers and combined factors in elderly participants.

| Variable | Cut-off value | Sensitivity | Specificity | *P* | AUC |
| --- | --- | --- | --- | --- | --- |
| Crea | 76.050 | 0.618 | 0.759 | 0.0006 | 0.7211 |
| ALT | 19.650 | 0.632 | 0.621 | 0.0187 | 0.6514 |
| GLU | 5.515 | 0.559 | 0.655 | 0.0669 | 0.6179 |
| Logistics | 0.6039703 | 0.779 | 0.724 | <0.0001 | 0.8174 |
